# Comparative analysis of emotional facial expression recognition and empathy in children with prader-willi syndrome and autism spectrum disorder

**DOI:** 10.1186/s40359-024-01590-3

**Published:** 2024-02-23

**Authors:** Ane Perosanz, Oscar Martínez, Patricia Espinosa-Blanco, Irune García, Mohammad Al-Rashaida, Juan Francisco López-Paz

**Affiliations:** 1 Faculty of Health Sciences, Department of Psychology, University of Deusto, Avenida de las Universidades, 24, 48007 Bilbao, Biscay, Spain; 2 College of Education, Department of Special Education, United Arab Emirates University, Al Ain, Abu Dhabi, United Arab Emirates

**Keywords:** Prader-Willi Syndrome, Autism spectrum disorder, Recognition of emotional facial expression, Empathy, Theory of mind

## Abstract

**Background:**

Prader-Willi Syndrome (PWS) is a rare neurodevelopmental disorder that is often comorbid with Autism Spectrum Disorder (ASD). Due to the close association between these two conditions, and recognizing that Theory of Mind (ToM) is related to social behaviors in ASD, there is a growing interest in studying the reciprocity of social communication between these two groups.

**Method:**

The primary objective of this study was to compare how children (*n* = 45) with PWS (*n* = 15), ASD (*n* = 15), and a control group (*n* = 15) respond to emotion recognition of facial expressions and empathy, which are both concepts related to ToM. The study utilized two tools named FEEL and Deusto-e-Motion 1.0. We also evaluated the Working Memory index of the WISC-IV scale, the Social Perception domain of the NEPSY-II battery, and the SCQ in both clinical groups.

**Results:**

Our findings suggest that individuals with PWS exhibit lower accuracy in recognizing facial expressions and empathy compared to the control group. Both clinical groups exhibited a delayed reaction time compared to the control group. Children with PWS display difficulties in recognizing emotions of disgust and surprise. In terms of cognitive empathy, children with PWS showed a greater inclination to respond to disgust as compared to children with ASD.

**Conclusions:**

This study represents the initial stage in comprehending the emotional and empathetic abilities of children with PWS and ASD. The findings can provide valuable insights for developing future interventions.

Autism Spectrum Disorder (ASD) is a neurodevelopmental disorder characterized by dysfunction in the central nervous system, resulting in impairments in communication and behavior [1]. The etiology of ASD involves a complex interplay of genetic and environmental factors that impact early brain development [2]. Over the past few decades, there has been a significant increase in the prevalence of ASD, especially in high-income countries such as the United States. The current diagnosis rate for ASD in the US is 1 in 54 children [3, 4]. In Europe, around 1 in 100 newborns are affected by ASD [5], which is a similar rate to what has been observed in Spain [6]. Notably, ASD is more frequently diagnosed in males, with a fourfold higher incidence compared to females. Diagnosis in girls and women can often be more challenging and delayed due to the subtler presentation of symptoms [3].

According to the 5th edition of the Diagnostic and Statistical Manual of Mental Disorders (DSM-5) of the American Psychiatric Association [7], the fundamental symptoms of ASD include persistent deficiencies in social interaction and communication. These deficits manifest as impairments in socio-emotional reciprocity, nonverbal communication for social interaction, and difficulties in establishing and maintaining relationships. Individuals with ASD exhibit restricted and repetitive patterns of behavior, as well as atypical sensory responses. These behavioral patterns include stereotyped or repetitive motor movements, insistence on sameness, inflexible adherence to routines, highly restricted interests, hyper- or hyporeactivity to sensory stimuli, and a preoccupation with sensory aspects of the environment [7].

Prader-Willi Syndrome (PWS) is a rare neurodevelopmental disorder that can be caused by various genetic abnormalities, including a deletion in chromosome 15 inherited from the father, maternal uniparental disomy (mUPD), an imprinting defect, or a chromosomal translocation [8, 9]. These various genetic mechanisms appear to affect the frequency and severity of the disease, as individuals with mUPD exhibit more severe symptoms [10, 11]. According to Heyman [12], there are between 350,000 and 400,000 people diagnosed with PWS worldwide. In Europe, the prevalence is estimated to be 1 in 50,000 inhabitants, with an annual incidence of 1 in 30,000 births [13]. In Spain, the incidence of the condition ranges from 1 in 15,000 to 1 in 25,000 newborns [14].

Physical manifestations of PWS include short stature, hypopigmentation, genital hypoplasia, and ocular abnormalities such as myopia and strabismus. Other relevant symptoms include hypotonia, exaggerated hyperphagia, and an increased risk of obesity during childhood and adolescence [15, 16]. Clinical manifestations of PWS include affective instability, obsessive-compulsive behaviors, and tantrums. Individuals with PWS may also present with mild to moderate intellectual disability [17]. Cognitive difficulties may manifest as issues with attention, mathematical skills, and working memory [18]. Additionally, delays in language acquisition are prevalent, with limitations in constructing semantic relationships, oral comprehension, and sentence formation [17].

Moreover, PWS is strongly linked to ASD, as 33% of individuals diagnosed with PWS also have ASD [19]. Individuals with PWS share certain behavioral symptoms with ASD, such as language disorders, perseverative thinking, and stereotypes [19]. Difficulties in social communication reciprocity are also observed in individuals with PWS. They have a poor ability to recognize and understand affective information, and struggle to understand others’ points of view. This has been related to the Theory of Mind (ToM) construct [20].

The ToM was first introduced by Premack and Woodruff [21] in 1978 as the ability to understand and interpret the behavior, thoughts, knowledge, and intentions of others. In 1985, Baron-Cohen et al. [22] applied the concept of the ToM to ASD, suggesting that deficits in ToM are a contributing factor to the symptoms exhibited by individuals with ASD. The ToM involves different levels of complexity, starting from the identification of emotions based on facial expressions and progressing to empathy and moral judgment [23].

The first component of ToM is facial emotional recognition, which involves the ability to deduce another person’s emotional state by analyzing their facial expressions [24]. Emotions can be classified as basic emotions, also called primary emotions (joy, disgust, fear, sadness, anger and surprise), which are considered innate, or secondary emotions [25]. The ability to recognize them in other people allows individuals to adapt to the social demands of the environment [26]. Authors such as Boccaccio et al. [27] and Fang et al. [28] argue that social, cultural, and psychological factors influence the ability to recognize specific emotions. Evidence seems to indicate that it is easier to recognize emotions when images depict the full face (whole face) rather than specific regions (eye or mouth region) [29]. Happiness is one of the easiest emotions to recognize in the whole face, while the most difficult is sadness [29, 30]. However, attending to all conditions (face, eyes and mouths) it seems that the most difficult emotion to recognize is surprise in children, fear in young elders and disgust in older adults [29].

Some studies suggest that children with PWS may struggle in social situations due to difficulties in attending to social cues, which are necessary for accurate perception of the social world. Recent research indicates that individuals with PWS have greater difficulty recognizing positive emotions compared to negative emotions [31, 32]. However, the findings related to facial emotional recognition in individuals with ASD are inconclusive. While some studies report no differences between individuals with ASD and the neurotypical population [33–35], others argue that children with ASD present difficulties in recognizing emotions through facial expression and intuiting other people’s thoughts [36–38], which hinders their social interactions and the development of prosocial behaviors.

Executive functions (EFs) are closely associated with ToM as both are located in the same brain region, the prefrontal cortex [39]. Children with traits of ASD often display deficiencies in both EFs and cognitive abilities, and there exists a correlation between their performance in these areas [40–43]. Studies have consistently shown that children with ASD demonstrate impairments in executive functioning, particularly in planning complex behaviors, which are related to deficits in working memory [44–46]. Individuals with this condition often experience difficulties with tasks that assess attention, cognitive flexibility, planning, inhibitory control, fluency, and working memory. These challenges can lead to perseverative behaviors and difficulty in changing strategies [44, 46]. However, they often perform better on tasks that involve visual-spatial abilities or pattern reproduction [44]. Children with PWS also face challenges in planning, problem-solving, working memory, inhibition, updating, and cognitive estimation tasks [17, 47]. Nevertheless, individuals with language impairments tend to exhibit preserved visuospatial development and reading ability [48, 49].

Empathy is defined as the ability to comprehend, be aware of, and be responsive to the emotions, thoughts, and experiences of others without them being communicated objectively or explicitly [50]. Empathy has been considered for so long one of the primary components of ToM [23, 51–53], however, Abdel-Hamid et al. [54] have recently suggested the opposite. In their study, they claim that they are two independent social cognition skills, so that an individual may have impaired ability to empathize, but not ToM deficits. The widely accepted model of empathy includes two systems: emotional empathy and cognitive empathy, which is similar to the ToM. Cognitive empathy refers to an individual’s capacity to recognize and comprehend another person’s perspective or emotional state without necessarily adopting it themselves [55]. On the other hand, emotional empathy involves an emotional response from an individual who is observing another person’s experience [56].

Although research on emotional and cognitive empathy in children with PWS is limited, some authors suggest that they may experience deficits in cognitive empathy as a result of a moderate delay in ToM [17, 20]. Recent studies suggest that individuals with ASD have deficits in cognitive empathy [57–59], but not emotional empathy [59]. These difficulties are associated with the functioning of the amygdala, the mirror neuron system, and the anterior insula at the neurological level [60]. However, there have been no studies comparing emotional and cognitive empathy in children with ASD and PWS.

Thus, taking into account that the literature on face recognition is very scarce in PWS and does not have a solid consensus in ASD, and as empathic processing has not yet been investigated comparatively between both syndromes, it seems necessary to investigate these aspects of psychosocial processing. Therefore, the main objective of the study is to carry out a comparative analysis of emotional facial expression recognition and empathy (both in terms of response accuracy and reaction time) among children with PWS, ASD and a control group. For this purpose, we will make a distinction between non-contextualized scenarios (correctly identifying a type of emotional facial expression) to assess facial recognition ability, and contextualized ones (associating an emotional facial expression with a virtual reality interaction) to assess empathic ability. On the other hand, taking into account that there is some comorbidity between both syndromes and that deficits in FFEE are frequent, in addition to analyzing tasks related to Emotion Recognition and ToM, Working Memory and ASD traits will also be evaluated, only between the clinical groups. This will establish a theoretical and empirical foundation for future research and the development of specific intervention designs.

## Methods

### Design

In this study, an ex post facto comparative design among children with PWS, ASD and a control group was used.

The evaluation of children with PWS was carried out both at the Deusto Psych Department of the University of Deusto or at children´s own homes, depending on each family preference. The evaluation of children with ASD took place at the Association of Parents of Children with Autism of Bizkaia (APNABI). The control group was evaluated in public and subsidized schools of the Basque Country that agreed to participate in the study.

### Participants

The sample consisted of a total of 45 boys and girls divided into three groups: a PWS group (*n* = 15), an ASD group (*n* = 15), and a control group (*n* = 15). The participants were paired based on age (*H* = 1.720, *p* = 0.423).

The PWS group comprised 10 boys (66.7%) and 5 girls (33.3%) who were diagnosed with PWS and ranged in age from 8 to 12 years (M = 9.47; SD = 1.36). All participants in this group (100%; *n* = 15) received growth hormone (GH) as part of their drug treatment. The diagnosis was confirmed by a genetic study (methylation analysis, classical cytogenetics, FISH techniques or microsatellite study by PCR) which confirms the alteration of chromosome 15 that characterizes PWS. It should be noted that none of them were diagnosed with ASD. Participants in this study were recruited from public child and adolescent mental health centers, such as Cruces University Hospital and Basurto University Hospital, as well as relevant Spanish associations, including the Spanish Association for PWS, the Catalan Association for PWS, and the Andalusian Association for PWS.

The ASD group consisted in 15 boys (100%) diagnosed with ASD, ranging in age from 8 to 12 years old (M = 10.07; SD = 1.36). The diagnosis of ASD was confirmed based on DSM-5 criteria. As with the previous clinical group, none of them were diagnosed with PWS. Participants in this group were recruited through APNABI Association.

Both the PWS and ASD groups were selected based on the following inclusion criteria: (1) a diagnosis of PWS or ASD, respectively; (2) an Intelligence Quotiente (IQ) range of 70–85 for borderline intelligence or an IQ range of 55–69 for mild intellectual disability as measured by the Wechsler Intelligence Scale for Children-IV (WISC-IV); (3) verbal language abilities (speaking, listening, reading and writing skills); and (4) familiarity with computer usage. Exclusion criteria were as follows: (1) age outside the range of 8 to 12; (2) diagnosis of psychotic disorders; (3) severe neurological symptoms that impeded proper test administration; (4) moderate or severe intellectual disability (IQ < 54); (5) lack of language skills; and (6) refusal to participate in the study.

The control group comprised 10 healthy boys (66.7%) and 5 girls (33.3%) aged between 8 and 12 years (M = 9.47; SD = 1.36). The control group participants were selected from a sample of over 1700 students who were used to validate the Facially Expressed Emotion Labeling (FEEL) and Deusto-e-Motion 1.0 instruments. The inclusion criteria for the control group were as follows: (1) completion of compulsory schooling, (2) enrollment in 3rd to 6th grade of primary education, and (3) proficiency in computer usage. Exclusion criteria comprised of the following: (1) a diagnosis of PWS, ASD, or any other psychiatric disorder; (2) presence of intellectual disability (IQ < 70); (3) undergoing psychiatric or psychological treatment; and (4) refusal to participate.

### Materials

The first instrument administered to the clinical sample was Form B of the Social Communication Questionnaire (SCQ) [61], which was given to parents and/or guardians. The SCQ assesses traits associated with ASD and is divided into three domains: Social, Communication, and Stereotypies. The maximum possible score is 39 if question 1 was “Yes” and 33 otherwise. As a general rule, 15 has been established as the cut-off point above which the existence of ASD is considered possible. The instrument has high reliability, with a reported alpha of 0.90 in the Spanish typification. Good discriminant validity was also reported in studies comparing samples with and without ASD (0.88) and those with ASD and intellectual disability (0.93) [62, 63].

The Working Memory Index of the Wechsler Intelligence Scale for Children-IV (WISC-IV) [64] was administered to evaluate the participant’s working memory abilities, that is, the ability to retain, store, transform and generate information. This index includes 3 tests: (1) Digits: analyzes the immediate memory and working memory, indicating sequencing skills, panning, alertness and cognitive flexibility; (2) Letters and Numbers: analyzes the ability to retain and combine two types of information, organize it and elaborate an organized set according to instructions; (3) Arithmetic: is optional and analyzes numerical reasoning skills, agility in handling and reorganizing information, attention and short-term memory. In the selected tests (Digits and Letters-Numbers), the total number of correct answers for each item is scored to obtain the direct score. These direct scores must be transformed into scalar scores (standard scores with M = 10; SD = 3) ranging from 1 to 19. The sum of the scalar scores of both tests gives the value of the MT index. The WISC-IV Manual [64] presents tables with which to obtain the percentile rank and confidence interval, as well as a brief interpretation of the MT index in terms of diagnostic categories (high, low, normal, within limits…). The Spanish version of the WISC-IV reports reliability coefficients ranging from 0.72 to 0.91. Specifically, for the selected tests, the reliability coefficient is 0.84. Concurrent validity was established by correlating the indices of the Wechsler Intelligence Scale for Children-III (WISC-III) and WISC-IV, which ranged from 0.72 to 0.89 [65].

The domain of Social Perception was assessed through the use of the NEPSY-II Child Neuropsychological Battery (NEPSY-II) [66], which comprises tasks designed to measure Emotion Recognition and Theory of Mind (ToM). The Emotion Recognition task assesses the ability to recognize emotions (joy, sadness, neutrality, fear, anger and disgust) in different photographs of boys and girls. The test score is the sum of the correct answers (ranging from 0 to 35). The ToM task is divided into two subtests: (1) verbal task: assesses the ability to understand the ideas, thoughts and beliefs of others, as well as figurative and imitative language. This task uses purely verbal items or items accompanied by a picture (subtest score ranges from 0 to 22); (2) contextual task: assesses the ability to understand and infer the relationship between emotions and social context (subtest score ranges from 0 to 6). Scores on both subtests were summed to calculate the total ToM score. The ToM task has a reliability coefficient of 0.99, while the Emotion Recognition task has a reliability coefficient of 0.78 for children aged 7 to 12 years based on Spanish norms. The construct validity demonstrated a very low correlation of 0.20 between the two tasks [67].

The Facially Expressed Emotion Labeling (FEEL) computer program [68] was used to evaluate the capacity to identify basic emotions based on static facial expressions. The program presents participants with 42 images of men and women displaying various emotional facial expressions. Participants are shown a neutral facial expression for 1.5 s, followed by an image displaying an emotional facial expression that they must identify within 300 milliseconds. The test has a maximum score of 42 points. The program has an application to encode the total number of correct answers, wrong scores and reaction times. The reliability coefficient of the test was reported as 0.77, based on a sample of over 400 participants [69].

The virtual tool Deusto-e-Motion 1.0, developed by Amayra et al. [70], was used to evaluate different variables associated with recognizing emotional expressions and empathic responses. The tool comprises three blocks: (1) 14 static emotional expressions and 10 dynamic emotional expressions based on a neutral expression. Participants are required to identify the corresponding emotion; (2) 6 static scenarios where participants identify the emotions experienced by the characters involved; and (3) 24 social situations that simulate a schoolyard. Participants are asked to identify their own emotions and the emotions of the characters involved. The Deusto-e-Motion 1.0 automatically records response accuracy, reaction time and response choice. For the evaluation of the results, it should be noted that data are automatically recorded during each application, and that these data generate percentiles based on age and gender. Tables indicating the percentiles for each of the blocks can be found in the Deusto-e-Motion 1.0 Manual [70]. The reliability coefficients for emotional facial recognition and reaction time were found to be medium to high, with values of 0.63 and 0.84, respectively. For reaction time in virtual settings, the reliability coefficient was 0.86 [70, 71].

### Procedure

For the clinical groups, we contacted the Department of Psychology of the relevant association or the child psychiatrist at the mental health center (for the PWS group) via phone. We sent an informative letter about the study via email, which was then forwarded to families. After obtaining their consent, we sent informed consent forms and agreed upon the evaluation dates. On the day of evaluation, families provided informed consent in compliance with the Psychologist’s Code of Ethics and Organic Law 15/1999 of December 13, which pertains to the protection of personal data. We conducted a brief initial interview with the parents and/or guardians to gather sociodemographic and clinical information about the child. We then administered Form B of the SCQ questionnaire. Subsequently, we assessed the children using the FEEL and Deusto-e-Motion 1.0 assessment tools. Finally, we administered the Social Perception Scale from the NEPSY-II battery and the Working Memory Index using the WISC-IV scale.

For the control group, we contacted public and subsidized schools in Basque Country by telephone and sent an informative letter about the study along with informed consent forms via email to the families. On the day of the evaluation, the families provided signed informed consent. We conducted a brief initial interview to gather sociodemographic information about minors. In this group, we administered only the FEEL and Deusto-e-Motion 1.0 instruments to the children. The procedures for obtaining informed consent and conducting the assessments were conducted in accordance with the ethical guidelines. This study was approved by the corresponding university ethics committee.

### Statistical analysis

All statistical analyses were conducted using IBM SPSS Statistics version 28.0.0.0 (190) for Windows. A *p* value (*p*) less than or equal to 0.05 was considered significant for a confidence level of 95%.

First, the normal distribution of all numerical variables in the sample was analyzed using the Kolmogorov-Smirnov test.

Second, descriptive statistics (mean, median, and standard deviation) were used to analyze clinical and demographic variables of a quantitative nature, whereas nominal variables were analyzed using frequency and percentage.

Third, the non-parametric Kruskal-Wallis H-test was used to analyze the differences among the three groups in the quantitative variables of response accuracy (total correct answers) in emotional recognition through facial expressions in non-contextualized scenarios and reaction times to said stimuli and before the contextualized variables. When statistically significant differences were obtained, pairwise multiple comparisons were performed using the post hoc Mann-Whitney U-test to determine between which two groups these differences appeared. Simultaneously, the Bonferroni correction (*p* divided by the number of comparisons) was applied, so the corrected significance level to be considered in the Mann-Whitney U-tests would be less than or equal to 0.017 (0.05/3). The *η2* (eta squared) [72] was calculated to measure the effect size for the Kruskal-Wallis test, where *η2* = 0.04 is considered minimum necessary, *η2* = 0.25 moderate and *η2* = 0.64 strong. Likewise, the *r* coefficient [73] was calculated to measure the effect size for the Mann-Whitney test, where *r* = 0.1 is considered small, *r* = 0.3 medium, and *r* = 0.5 large.

Fourth, the frequency of responses each group gave to the nominal variables of emotion recognition in contextualized scenarios was observed using a chi-squared statistical test.

Fifth, statistically significant differences in Emotion Recognition, ToM, Working Memory, and ASD trait variables were analyzed using the Mann-Whitney U test for two independent samples (PWS and ASD).

Finally, the variables of total correct answers and total reaction time in the recognition of emotional facial expressions were analyzed using Spearman’s Rho non-parametric correlation tests. Correlations were only examined between the PWS and ASD groups for Emotion Recognition, ToM, Working Memory, and ASD traits.

## Results

The results of the study indicate the statistically significant differences obtained among the three groups in the variables of recognition of emotional facial expressions in both non-contextualized and contextualized scenarios. In addition, only among the clinical groups, the statistical differences in the variables of Emotion Recognition, ToM, Working Memory and ASD traits are presented. Finally, a correlation analysis between all these variables is presented.

### Emotional facial expression recognition variables and empathy

#### Non-contextualized scenarios

Regarding the FEEL tool, the Kruskal-Wallis H-test analysis for the three groups indicated statistically significant differences in response accuracy in the total correct answers for facial expressions (*H* = 9.740; *p* = 0.008; *η2* = 0.184), particularly in correctly identifying surprise (*H* = 13.964; *p* < 0.001; *η2 =* 0.285) and disgust (*H* = 9.816; *p* = 0.007; *η2 = 0.*186). Differences were found in the reaction time for emotions of fear (*H* = 21.969; *p* < 0.001; *η2 =* 0.475), happiness (*H* = 10.973; *p* = 0.004; *η2 = 0.*214), surprise (*H* = 21.894; *p* < 0.001; *η2 = 0.*474), disgust (*H* = 25.659; *p* < 0.001; *η2 = 0.*563), sadness (*H* = 15.384; *p* < 0.001; *η2 =* 0.319), anger (*H* = 24.720; *p* < 0.001; *η2 =* 0.541) and total (*H* = 23.523; *p* < 0.001; *η2 =* 0.512).

The post hoc Mann-Whitney U-test indicated a higher response accuracy and a shorter reaction time by the control group in contrast to the clinical groups. The PWS group presented a longer reaction time than the ASD group in the emotions of fear, happiness, surprise and total, and a shorter reaction time in disgust (Table 1).

**Table 1:** Response accuracy and reaction time of the FEEL tool with statistically significant differences by pairs

	Emotions	Control (n = 15) M ± SD	PWS (n = 15) M ± SD	ASD (n = 15) M ± SD		*U*		**z**	*p*	*r*	Post Hoc
**Response accuracy**											
	Total	30.95 ± 5.27	21.95 ± 7.35	4.33 ± 2.41		34.000		-3.256	0.001	0.59	Control > PWS
	Surprise	5.93 ± 1.28	2.93 ± 2.20	4.33 ± 2.53		22.000		-3.829	< 0.001	0.69	Control > PWS
	Disgust	4.93 ± 2.34	2 ± 1.51	3.33 ± 2.69		37.500		-3.139	0.002	0.57	Control > PWS
**Reaction time**											
	Total	3382.61 ± 1520.23	9950.83 ± 11254.90	3684.10 ± 1954.78		9.000		-4.293	< 0.001	0.78	Control < ASD
						15.000		-4.044	< 0.001	0.74	PWS > ASD
	Fear	4432.27 ± 3119.22	15279.83 ± 27885.52	3787.08 ± 2039.02		11.000		-4.210	< 0.001	0.77	PWS > ASD
						20.000		-3.837	< 0.001	0.70	Control < PWS
	Happiness	2604.96 ± 1144.44	9686.29 ± 20933.67	3391.05 ± 2684.89		7.000		-4.376	< 0.001	0.79	Control < PWS
						56.000		-2.344	0.015	0.43	PWS > ASD
	Surprise	3124.39 ± 1534.57	10319.22 ± 8064.15	4004.01 ± 2491.65		26.000		-3.588	< 0.001	0.65	PWS > ASD
	Disgust	3389.26 ± 2154.17	8900.09 ± 2915.69	3792.13 ± 2391.78		3.000		-4.542	< 0.001	0.83	PWS < ASD
						12.000		-4.169	< 0.001	0.76	Control < PWS
	Sadness	3275.53 ± 1316.72	6834.71 ± 3720.31	3925.78 ± 2626.57		21.000		-3.795	< 0.001	0.69	Control < PWS
						43.000		-2.883	0.004	0.57	PWS > ASD
	Anger	3529.63 ± 1411.14	8672.11 ± 5323.74	3204.57 ± 1987.54		9.000		-4.293	<0.001	0.78	Control < PWS
							11.000	-4.044	< 0.001	0.74	PWS > ASD

n: number of participants; M: mean; SD: standard deviation; U: Mann-Whitney U-test; z: standard scored; *p*: statistical probability *p*-value; r: effect size

Bonferroni post hoc analysis with corrected significance level *p* ≤.017

Regarding the Deusto-e-Motion 1.0 virtual reality tool (Block 1), statistically significant differences were identified among all groups in the number of correct responses for fear (*H* = 9.997; *p* = 0.007; *η2 =* 0.190) and the reaction times for static faces (*H* = 7.974; *p* = 0.019; *η2 = 0.*142), fear (*H* = 6.239; *p* = 0.044; *η2 =* 0.101), happiness (*H* = 7.071; *p* = 0.029; *η2 =* 0.121) and anger (*H* = 6.422; *p* = 0.040; *η2 =* 0.105).

Using the post hoc Mann-Whitney U-test, it was found that the PWS group had a lower response accuracy than the ASD group in the recognition of the emotion of fear. It was also found that the control group had a shorter reaction time than the PWS group in static faces and fear, and a shorter reaction time than the ASD group in happiness and disgust (Table 2).

**Table 2:** Response accuracy and reaction time of block 1 of the Deusto-e-Motion 1.0 (non-contextualized scenarios) with statistically significant differences in pairs

	Emotions	Control (*n* = 15) M ± SD	PWS (*n* = 15) M ± SD	ASD (*n* = 15) M ± SD	***U***	***z***	***p***	***r***	Post Hoc
**Response accuracy**									
	Fear	1.47 ± 0.92	0.60 ± 0.91	1.87 ± 1.19	47.000	-2.877	0.004	0.53	PWS < ASD
**Reaction time**									
	Static faces	5745.49 ± 1309.84	8351.95 ± 285.67	9188.76 ± 4999.39	41.000	-2.966	0.003	0.54	Control < PWS
	Fear	8328.17 ± 10140.45	8425.74 ± 1864.64	8924.44 ± 7980.27	50.000	-2.593	0.010	0.47	Control < PWS
	Happiness	6204.92 ± 2171.41	8347 ± 5214.62	10359.33 ± 4597.12	46.000	-2.758	0.006	0.50	Control < ASD
	Anger	4732.57 ± 1272.06	7209.33 ± 3637.76	8304.67 ± 4867.85	59.000	-2.219	0.015	0.40	Control < ASD

n: number of participants; M: mean; SD: standard deviation; U: Mann-Whitney U-test; z: standard scored; **p**: statistical probability *p*-value; r: effect size

Bonferroni post hoc analysis with corrected significance level *p* ≤.017

Table 3 presents the means and standard deviations of the variables related to the recognition of emotional facial expressions assessed using the FEEL and Deusto-e-Motion 1.0 tools.

**Table 3:** Means and standard deviations of the recognition of emotional facial expression variables (response accuracy and reaction time)

	Control (*n* = 15) M ± SD	PWS (*n* = 15) M ± SD	ASD (*n* = 15) M ± SD
**Response accuracy**			
Total	47.83 ± 5.52	36.88 ± 9.95	42.91 ± 17.75
Fear	5.67 ± 2.41	3.47 ± 2.03	6.20 ± 3.36
Happiness	10.33 ± 1.05	10.00 ± 2.33	8.27 ± 2.94
Surprise	8.87 ± 2.53	4.73 ± 2.49	6.47 ± 3.50
Disgust	7.07 ± 2.31	3.73 ± 2.05	5.40 ± 3.62
Sadness	8.20 ± 2.04	8.00 ± 2.65	7.73 ± 4.03
Anger	7.73 ± 2.55	6.07 ± 2.40	7.07 ± 3.49
Neutral	1.80 ± 0.86	1.53 ± 1.06	1.80 ± 1.32
**Reaction time**			
Total	12880.91 ± 11767.83	30753.92 ± 52902.55	12652.42 ± 6548.58
Fear	12760.44 ± 13259.67	23704.99 ± 29750.16	12711.52 ± 10019.29
Happiness	8809.81 ± 3315.85	18033.29 ± 26150.29	13750.38 ± 7282.06
Surprise	8484.27 ± 3341.12	17992.53 ± 10432.68	13010.68 ± 9046.13
Disgust	10002.98 ± 3758.12	18024.51 ± 5249.83	22293.94 ± 47661.79
Sadness	11801.18 ± 14578.19	14183.37 ± 6010.42	10067.78 ± 7129.58
Anger	8262.04 ± 2683.20	15881.44 ± 8961.50	11509.24 ± 6855.39
Neutral	8709.41 ± 3124.98	15880.96 ± 18900.35	13196.44 ± 5577.05

M: mean; SD: standard deviation

Note: neutral emotion only evaluated through the Deusto-e-Motion 1.0 virtual tool

#### Contextualized scenarios

A frequency analysis (refer to Table 4) was conducted using the chi-square test to examine the qualitative trends in emotional responses to contextualized emotion items in Deusto-e-Motion 1.0 (Blocks 2 and 3). Statistically significant differences were observed among the three study groups for certain contextualized items (see Table 5).

**Table 4:** Response accuracy of blocks 2 and 3 of the Deusto-e-Motion 1.0 (contextualized scenarios) with statistically differences among the control group, PWS and ASD.

Items	Descriptor of the contextual scene	${\chi ^2}$	***V***	***p***
8.2	Obedience status	23.764	0.514	0.049
10.1	Unexpected event situation	10.755	0.346	0.029
14.1	Situation of social exclusion of people with functional diversity	16.727	0.431	0.033
14.2	Situation of social exclusion of people with functional diversity	23.565	0.512	0.009
17	Social inclusion situation	24.306	0.520	0.007
23.1	Sharing situation	18.306	0.451	0.019
24.2	Puzzling situation	19.657	0.467	0.033
25.1	Exhaust situation	26.631	0.544	0.009
25.2	Exhaust situation	40.188	0.668	< 0.001

${\chi ^2}$: Chi-Square; V: Cramer´s V; p: statistical probability *p*-value

**Table 5:** Frequencies of the subjective responses of blocks 2 and 3 of the deusto-e-motion 1.0 (contextualized scenarios) with statistically significant differences among the control group, PWS and ASD

						Items				
Emotions	Groups	8.2	10.1	14.1	14.2	17	23.1	24.2	25.1	25.2
Fear	Control	50%			0%		35.5%	0%	0%	7.1%
	PWS	50%			33.3%		41.9%	0%	22.2%	35.7%
	ASD	0%			66.7%		22.6%	100%	77.8%	57.1%
Happiness	Control	10%		50%	10%	6.3%		0%	37.5%	90.9%
	PWS	60%		50%	80%	56.3%		0%	25%	0%
	ASD	30%		0%	10%	37.5%		100%	37.5%	9.1%
Surprise	Control	0%	25%			66.7%	75%		33.3%	0%
	PWS	100%	20%			22.2%	0%		33.3%	50%
	ASD	0%	55%			11.1%	25%		33.3%	50%
Disgust	Control	0%		100%				0%	64.3%	0%
	PWS	100%		0%				100%	35.7%	85.7%
	ASD	0%		0%				0%	0%	14.3%
Sadness	Control	25%	66.7%		53.8%	42.9%	50%	40%	0%	0%
	PWS	62.5%	0%		0%	0%	50%	26.7%	100%	50%
	ASD	12.5%	33.3%		46.2%	57.1%	0%	33.3%	0%	50%
Anger	Control	47.6%			12.5%	16.7%	0%	14.3%	0%	0%
	PWS	4.8%			50%	66.7%	33.3%	85.7%	50%	50%
	ASD	47.6%			37.5%	16.7%	66.7%	0%	50%	50%
Neutral	Control	0%			50%	40%	0%	50%	33.3%	75%
	PWS	0%			20%	0%	0%	0%	0%	0%
	ASD	100%			30%	60%	100%	50%	66.7%	25%

Similarly, statistically significant differences were observed in the reaction time of certain contextualized emotion items in Deusto-e-Motion 1.0 (Blocks 2 and 3) among all groups. The post hoc Mann-Whitney U-test showed a shorter reaction time by the control group compared to the clinical groups, and differences in terms of longer or shorter reaction time between the clinical groups in some contextualized situations (Table 6).

**Table 6:** Response time of blocks 2 and 3 of the Deusto-e-Motion 1.0 (contextualized scenarios) with statistically significant differences among three groups and in pairs

		Differences among 3 groups				Pairwise differences				
Items	Descriptor of the contextual scene	***H***	η2	***p***		***U***	z	***p***	***r***	Post Hoc
10.1	Unexpected event situation	6.073	0.097	0.048		53.000	-2.468	0.014	0.45	Control < ASD
12	Situation of social inclusion of people with functional diversity	14.202	0.291	< 0.001		33.000 39.00	-3.298 -3.049	< 0.001 0.002	0.60 0.56	Control < PWS Control < ASD
14.2	Situation of social exclusion of people with functional diversity	18.490	0.393	< 0.001		37.000 16.000	-3.298 -4.003	0.002 < 0.001	0.60 0.73	Control < PWS Control < ASD
16	Unexpected event situation	10.361	0.199	0.006		60.000 36.500	-2.178 -3.279	0.016 0.001	0.39 0.59	Control < ASD PWS > ASD
17	Social inclusion situation	13.367	0.271	0.001		30.000 52.500	-3.423 -2.492	< 0.001 0.013	0.62 0.45	Control < ASD PWS > ASD
18	Frustration tolerance	7.195	0.124	0.027		52.500	-2.489	0.013	0.45	PWS > ASD
19.1	Frustration tolerance	11.283	0.221	0.004		44.500 42.000	-2.822 -2.925	0.005 0.003	0.51 0.53	Control < PWS Control < ASD
19.2	Frustration tolerance	7.480	0.130	0.024		50.500	-2.572	0.010	0.47	PWS < ASD
20.1	Sharing situation	8.154	0.147	0.017		46.500	-2.739	0.006	0.49	PWS < ASD
22.1	Interpersonal conflict situation	17.750	0.375	< 0.001		16.000	-4.003	< 0.001	0.73	Control < ASD
22.2	Interpersonal conflict situation	7.128	0.122	0.028		50.000	-2.593	0.010	0.47	Control < ASD
23.1	Sharing situation	7.603	0.133	0.022		52.000	-2.513	0.012	0.46	PWS > ASD
25.2	Exhaust situation	6.185	0.100	0.045		56.000	-2.344	0.016	0.43	Control < ASD
27	Obedience status	11.853	0.235	0.003		36.500	-3.153	0.002	0.58	PWS > ASD

H: Kruskal-Wallis H-test; η2: (eta squared) effect size for Kruskal-Wallis; U: Mann-Whitney U-test; z: standard scored; r: effect size for Mann-Whitney; *p*: statistical probability *p*-value

Significance level *p* ≤.05 for differences among three groups

Bonferroni post hoc analysis with corrected significance level *p* ≤.017 for pairwise differences

### Emotion recognition, ToM, working memory, and ASD trait variables between clinical groups

In terms of the Emotion Recognition and ToM variables from the NEPSY-II battery, the Working Memory Index of the WISC-IV scale, and the Social, Communication, and Stereotypy domains of the SCQ questionnaire, significant differences were observed between clinical groups (PWS and ASD) using the Mann-Whitney U test with two independent samples. Specifically, significant differences were found in the Emotion Recognition (*U* = 55.500; *z*=-2.384; *p* = 0.017; *r* = 0.44) and ToM tasks (*U* = 48.000; *z*=-2.740; *p* = 0.006; *r* = 0.50) of the NEPSY-II, as well as in the Social domain (*U* = 62.500; *z*=-2.090; *p* = 0.037; *r* = 0.38) of the SCQ.

### Correlation analysis between variables

Finally, correlation analyses were conducted using Spearman’s Rho non-parametric tests. Positive and statistically significant correlations were observed between Emotion Recognition and ToM variables of the NEPSY-II battery (*Rho* = 0.434; *p* = 0.017). Additionally, significant correlations were found between different domains of the SCQ: Social and Communication (*Rho* = 0.493; *p* = 0.006), Social and Stereotypes (*Rho* = 0.593; *p* < 0.001), and Communication and Stereotypes (*Rho* = 0.374; *p* = 0.042). Furthermore, negative correlations were found between the Emotion Recognition score of the NEPSY-II and the Communication domain of the SCQ (*Rho*=-0.407; *p* = 0.025) as well as between the ToM score of the NEPSY-II and the total score of the SCQ (*Rho*=-0.420; *p* = 0.021) and the communication domain of the SCQ (*Rho*=-0.384; *p* = 0.036). Finally, significant positive correlations were found between the total correct answers in the FEEL and Deusto-e-Motion 1.0 tests (*Rho* = 0.655; *p* < 0.001) and between the total reaction time in both tests (*Rho* = 0.533; *p* < 0.001). Negative correlations were observed between the total rection time in the FEEL test and the total correct answers in both the FEEL (*Rho*=-0.605; *p* < 0.001) and Deusto-e-Motion 1.0 (*Rho*=- 0.361; *p* = 0.015) tests. Additionally, a negative correlation was found between the total reaction time in Deusto-e-Motion 1.0 and the total correct answers in the FEEL test (*Rho*=-0.414; *p* = 0.005).

## Discussion

The results of this study showed that both clinical groups (PWS and ASD) had longer reaction times than the control group when recognizing emotional and empathic facial expressions. Likewise, the PWS group provided less precise answers compared to the control. However, there were no statistically significant differences between the ASD and control groups in the correct recognition of emotional facial expressions, although there were differences in cognitive empathy in situations of unexpected events, social exclusion of people with functional diversity, sharing experiences, and exhaustion.

Statistically significant differences in response accuracy were observed among the three groups. Specifically, differences were found between the control group and PWS in the emotions of surprise and disgust, indicating that individuals with PWS may have difficulty recognizing emotions through facial expressions, as previously reported by Dykens et al. [31] and Famelart et al. [32]. However, no significant differences in response accuracy were observed between the control and ASD groups. These results are consistent with other evidences showing that children and adults with ASD can recognize basic emotions with equal or better accuracy than the control group [74–76]. This contradicts the view that individuals with ASD present deficits in emotional recognition [37, 77, 78]. Statistically significant differences between the clinical groups were found in the emotion of fear, which could be due to the deficit in detecting terror shown by children with ASD, as reported by Howard et al. [79] and Ruggieri & Arberas [80].

Most differences in emotional response accuracy were observed for the items evaluated using the FEEL tool. This test appears to discriminate performance in these variables more precisely than the Deusto-e-Motion 1.0 tool, as the virtual reality test includes 24 emotion recognition items [70] compared to 42 items with photographs of real faces in the FEEL test [68]. However, Deusto-e-Motion 1.0 includes 10 items of dynamic emotional expressions based on a neutral expression, although no statistically significant differences were found between any of the study groups in this condition.

Statistically significant differences were also observed among the three groups when reaction time was analyzed. Children with ASD required significantly more time than the control group, which may be due to the difficulties in attention [44], the alterations in cognitive flexibility, or the tendency to perseverate that they have [46]. Children with ASD also respond more quickly to stimuli containing negative cues, such as expressions of fear, anger and sadness, than positive cues, such as joy [81]. Similarly, the PWS group reacted more quickly to stimuli that contained negative cues, such as emotions of disgust, sadness, and anger, than positive ones, like joy, which aligns with what was previously reported by Dykens et al. [31]. When comparing the clinical groups, statistically significant differences were found, but only in the FEEL test, which requires reading emotions to choose the label corresponding to the face presented. In contrast, most of the verbal responses in the Deusto-e-Motion 1.0 tool were accompanied by visual support (image of the emotion). Therefore, it appears that language difficulties in both groups may be interfering, and that the differences are not solely due to motor-visual-perceptive skills of children with PWS [82].

The three study groups differed significantly in the types of responses given to the contextualized items. These differences were primarily observed in items that assessed cognitive empathy, which requires more advanced mentalization skills involving understanding the situation and inferring or intuiting the emotions of others. Some children with PWS and ASD have greater difficulty recognizing emotions in contextualized scenarios, and consequently, in cognitive empathy tasks. These findings are consistent with previous research by Castilla Ortiz [57] and Mul et al. [59], who observed deficits in cognitive empathy among individuals with ASD. These results are also consistent with the studies by Guinovart et al. [17] and Lo et al. [20], who found similar difficulties in the PWS population. The PWS group exhibited a higher frequency of subjective responses to the emotions of joy and disgust, whereas the ASD group did not react to the emotion of disgust and demonstrated a higher frequency of responses to the neutral emotion. This suggests that children with PWS may be more inclined to respond to the emotion of disgust than children with ASD in cognitive empathy tasks. Significant differences were also found in the reaction time of the contextualized scenarios among all groups. The clinical population groups exhibited significantly longer reaction times than the control group in situations involving social inclusion and exclusion of individuals with functional diversity as well as frustration tolerance.

No statistically significant differences in working memory were found between the clinical groups. However, statistically significant differences were observed in the NEPSY-II Emotion Recognition task between both. The consistent and systematic interventions provided to ASD patients in the APNABI Association may have influenced their performance in this task compared to the PWS group. Additionally, individuals with PWS demonstrated better performance in tasks that relied on perceptual cues for emotional recognition, such as learning through videos and illustrations [82], as opposed to tasks focused on emotional labeling. Likewise, significant differences were found in the ToM task of the NEPSY-II battery, possibly attributable to the average delay of four years in ToM skills observed in individuals with PWS [17]. Finally, statistically significant differences were also observed in the Social domain of the SCQ between both, indicating that children with ASD present greater difficulties in social interactions than children with PWS.

The results of this study did not show a statistically significant correlation between the WISC-IV Working Memory Index and NEPSY-II ToM task. This contradicts previous research, which found a positive correlation between these two domains [83, 84]. However, a statistically significant negative correlation was found between the total score on the SCQ and the ToM task of the NEPSY-II battery. This finding is consistent with the work of Losh et al. [85], who also found that ASD traits were associated with poorer performance on ToM tasks.

In addition, a statistically significant and positive correlation was found between the total correct answers in the Deusto-e-Motion 1.0 and FEEL tests. This is consistent with the validation results of both the tests by Lázaro et al. [71]. Similarly, a statistically significant and negative correlation was found between the reaction time in the FEEL test and the total correct answers in both FEEL and Deusto-e-Motion 1.0, and between the reaction time in the Deusto-e-Motion 1.0 test and the total correct answers in the FEEL. This indicates that the responses were not random, and that faster reaction times were associated with lower accuracy.

This study has several limitations. First, the FEEL and Deusto-e-Motion 1.0 tools have not been specifically validated for use in populations with intellectual disabilities or borderline intelligence quotient. Therefore, caution should be exercised when generalizing these findings to individuals with such characteristics. Additionally, the study had a relatively small sample size, particularly when controlling for age as a variable. A larger and more diverse sample size would enhance the generalizability of the results. Another limitation is that the ASD sample consisted exclusively of males. This gender bias could potentially influence the research findings, as evidence suggests that there are differences in the behavioral phenotypes of boys and girls with ASD [7].

## Conclusion

This study evaluated empathic and emotional facial expression recognition skills among children with PWS, ASD and a control group. The results showed that the PWS group had lower response accuracy in emotional facial expression recognition and empathy than the control group. Longer reaction times were also found in both clinical groups (PWS and ASD) versus the control. In general, children with PWS presented deficits in the recognition of the emotions of disgust and surprise. In addition, children in the clinical groups reacted faster to stimulus containing negative than positive cues. Some children with PWS and ASD experienced difficulties in cognitive empathy tasks. Specifically, children with PWS showed a greater tendency to respond to the emotion of disgust than children with ASD in this type of task. Regarding the analysis of working memory and ASD symptomatology, children with ASD were found to have greater difficulties in social interactions than children with PWS.

In summary, the results of the study provide valuable information for the development of new interventions to facilitate a better psychosocial adjustment in children with PWS and ASD.

## Data Availability

The data set generated and analyzed during the current study are not publicly available because they belong to the University of Deusto, but are available from the corresponding author upon reasonable request.

## Abbreviations

PWS: Prader-Willi Syndrome
ASD: Autism Spectrum Disorder
DSM-5: 5th edition of the Diagnostic and Statistical Manual of Mental Disorders
mUPD: Maternal uniparental disomy
ToM: Theory of Mind
EFs: Executive functions
APNABI: Association of Parents of Children with Autism of Bizkaia
IQ: Intelligence Quotiente
WISC-IV: Wechsler Intelligence Scale for Children-IV
FEEL: Facially Expressed Emotion Labeling
SCQ: Social Communication Questionnaire
WISC-III: Wechsler Intelligence Scale for Children-III
NEPSY-II: NEPSY-II Child Neuropsychological Battery
FISH: Fluorescence in situ hybridization
PCR: Polymerase chain reaction
EI: Emotional Intelligence
n: Number of participants
M: Mean
SD: Standard deviation
H: Kruskal-Wallis H-test
p: Statistical probability *p*-value
η2: Eta squared (effect size for Kruskal-Wallis)
U: Mann-Whitney U-test
z: Stadard scored
r: Effect size for Mann-Whitney
$${\chi ^2}$$: Chi-Square
V: Cramer´s V
Rho: Spearman’s Rho test
